# Continuous dynamic microforce reconstruction using electrical stimulation for remote pulse diagnosis

**DOI:** 10.1016/j.fmre.2025.09.002

**Published:** 2025-09-09

**Authors:** Xiaowei Zhao, Yuxin Song, Bojing Shi, Yubo Fan

**Affiliations:** Key Laboratory for Biomechanics and Mechanobiology of Ministry of Education, Beijing Advanced Innovation Center for Biomedical Engineering, School of Biological Science and Medical Engineering, School of Engineering Medicine, Beihang University, Beijing 100191, China

**Keywords:** Electrotactile, Tactile reconstruction, Pulse conditions, Remote diagnosis, Electric stimulation

## Abstract

Traditional Chinese medicine (TCM) pulse diagnosis has a long history, characterized by doctors forming subjective diagnostic opinions in their minds based on the patient’s pulse felt with their fingertips, rather than simply diagnosing based on pulse waves. As a result, TCM pulse diagnosis is significantly limited by spatial location, meaning that doctors and patients need face-to-face consultations, making remote diagnosis difficult. To address this spatial limitation, we propose a pulse reconstruction scheme based on the principle of electrotactile stimulation, allowing a remote feeling of pulses through electrical stimulation of the fingertips. We develop a simple system that uses electrical stimulation circuits and flexible fingertip electrodes, enabling doctors to remotely “touch” the patient’s pulse by placing their fingers on these electrodes. We investigate the relationship between electrical stimulation settings and tactile sensations, determining optimal ranges (1–6 mA current, 100 V voltage, 100–800 μs pulse width, and 100 pulses per cycle) that mimic a tactile pressure of 0.17–1 N. Additionally, wearable sensors capture pulse signals, converting them into electrical parameters that our device uses to recreate pulse sensations through electrical stimulation. The results indicate that remote pulse reconstruction through the electrotactile approach is feasible, providing a reference for remote TCM pulse diagnosis.

## Highlights

• Electrotactile-based pulse wave reconstruction: the technology leverages electrotactile principles to mechanically reconstruct human pulse waves, enabling remote pulse diagnosis with high fidelity.
• Mathematical modeling and feature-based waveform definition: pulse conditions are precisely defined using distinctive features and mathematical models, allowing for accurate and standardized waveform analysis.
• Dynamic pulse wave transformation and tactile reconstruction: the system explores the relationship between electrical stimulation parameters and force presentation, facilitating the conversion of pulse wave electrical signals into dynamic pulsations. Additionally, the tactile reconstruction device can replicate any pulse waveform, offering versatility and convenience in pulse diagnosis.

## Graphical abstract

**Figure:**
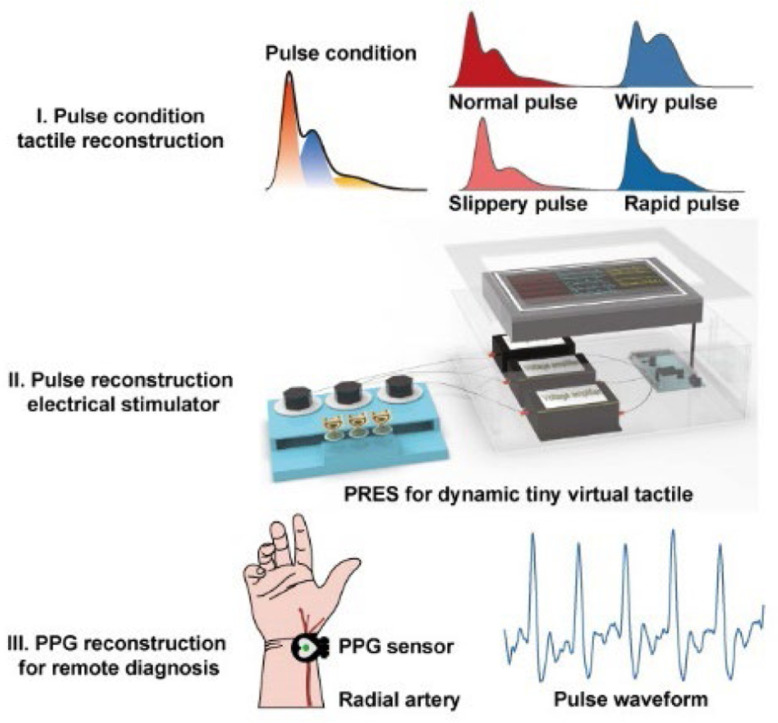

## 1 Introduction

Pulse diagnosis, an integral component of traditional Chinese medicine (TCM), utilizes varying finger pressures to palpate the radial artery and discern the patient’s physiological condition [1,2]. The wrist pulse results from the aortic wall's elastic relaxation and contraction, which is triggered by the ejection of blood from the heart, leading to the fluctuation of blood flow. Alterations in the health status of the human body can be manifested through changes in hemodynamics within the circulatory system at the radial artery [[3], [4], [5]]. Therefore, in TCM, doctors often take pulses at three positions of the radial artery to diagnose physical conditions, known as Cun, Guan, and Chi, according to *The Classic of Difficult Issues* (Nan Jing) [[6], [7], [8]]. The illustration of the locations of Cun, Guan, and Chi can be found in Fig. S1. Pulse states are categorized into 28 distinct pulse conditions, comprising a normal pulse and an additional 27 abnormal pulses. The normal pulse, also called the common pulse, indicates the pulse of a healthy individual. The remaining 27 pulse conditions, enumerated in Table 1, encompass diverse characteristics and have already been employed in clinical applications [9]. More details of pulse diagnosis and pulse conditions are explained in Supporting Information [10,11].

**Table 1:** **Characteristics of TCM pulse-conditions**.

Characteristic	Pulse conditions
Depth (Finger force)	Floating, deep, hidden
Strength and trend	Faint, weak, feeble, full
Tensity and shape (Fluid qualities)	Tight, taut, tympanic, wiry, hollow, soggy, slippery, rough, bouncing
Width and length	Surge, thready, long, short
Pulse rate and rhythm	Slow, rapid, racing, running, knotted, intermittent, scattered

Although there have been some studies utilizing sensors and computers for measuring and analyzing pulse signals [12], the TCM pulse diagnosis relies heavily on the subjective perception of the pulse tactile information by TCM doctors. A significant feature of pulse diagnosis is that doctors need to touch the patient’s radial artery and analyze the pulse based on their own experience to make a disease diagnosis. To further stimulate the effectiveness of this thousand-year-old pulse diagnosis technology, many scholars have researched how to achieve remote pulse diagnosis, which primarily relies on mechanical methods such as hydraulic systems, airbag systems, and linear motors [13,14]. To present pulse mechanics, researchers have developed a mock circulatory platform that can reconstruct blood pressure based on the Windkessel model to provide a palpation experience based on human cardiovascular physiology [15]. Linear motors are used to reconstruct the radial sense of touch, and a “Hap-pulse” haptic glove employs a fourth-order polynomial to fit various types of photoplethysmography (PPG) curves representing different pulse waves [16].

It is worth noting that some methods and devices involved in virtual reality technology are expected to achieve the reconstruction of small dynamic forces similar to pulses. Many research papers have proposed virtual tactile as a haptic reconstruction method that uses mechanical or electrical devices [[17], [18], [19], [20]]. These devices can generate corresponding tactile information, such as vibrations and pressure, by simulating various systems, primarily for applications in human-computer interaction and virtual reality [[21], [22], [23]]. The simulation of the human circulatory system has received increasing attention in recent years. The tactile reconstruction of arterial pulsation, specifically in the radial and carotid arteries within the human circulatory system, can serve as a valuable tool for training medical students and scientific research in pulse diagnosis [24]. Mechanical tactile feedback systems, which rely on motor, hydraulic, or pneumatic systems to drive physical components, are known for their realistic tactile sensations but suffer from significant drawbacks such as high energy consumption, large size, and slow response speed. In contrast, electrotactile reproduction techniques offer a more efficient and versatile alternative. By directly stimulating the skin with electrical currents, electrotactile systems consume minimal power, making them ideal for portable and battery-powered applications. Their fast response time ensures real-time tactile feedback, enhancing user immersion in virtual reality and augmented reality experiences. Additionally, electrotactile devices are compact and easily wearable, and they can generate a wide range of tactile sensations through adjustable parameters, making them suitable for diverse applications from medical rehabilitation to consumer electronics. These advantages make electrotactile reproduction a superior choice for applications demanding high efficiency, quick response, and versatility, while also addressing the limitations of traditional mechanical systems.

The generation of human tactile sensation depends on mechanoreceptors and nerve endings in the dermis of the skin. Electrotactile is generated by directly applying an electric current to the skin, stimulating mechanical receptors and nerve fibers. Specifically, electrotactile involves the application of electrical current to the human skin via surface electrodes, aiming to activate mechanoreceptors responsible for converting mechanical stimuli into action potentials. These action potentials are then conducted and transmitted to the central nervous system, resulting in the perception of tactile sensation [25]. Electrotactile can reconstruct pressure, vibration, tingling, and other haptics [[26], [27], [28], [29]]. And the type of tactile sensations can be changed by electrical stimulation parameters, such as frequency, pulse width, and pulse amplitude [30]. The application of electrotactile technology spans various domains, including virtual reality, augmented reality, gaming, and the medical field [[31], [32], [33]]. The electrotactile theory is explained in the Supporting Information and Fig. S2.

**Fig. 1:**
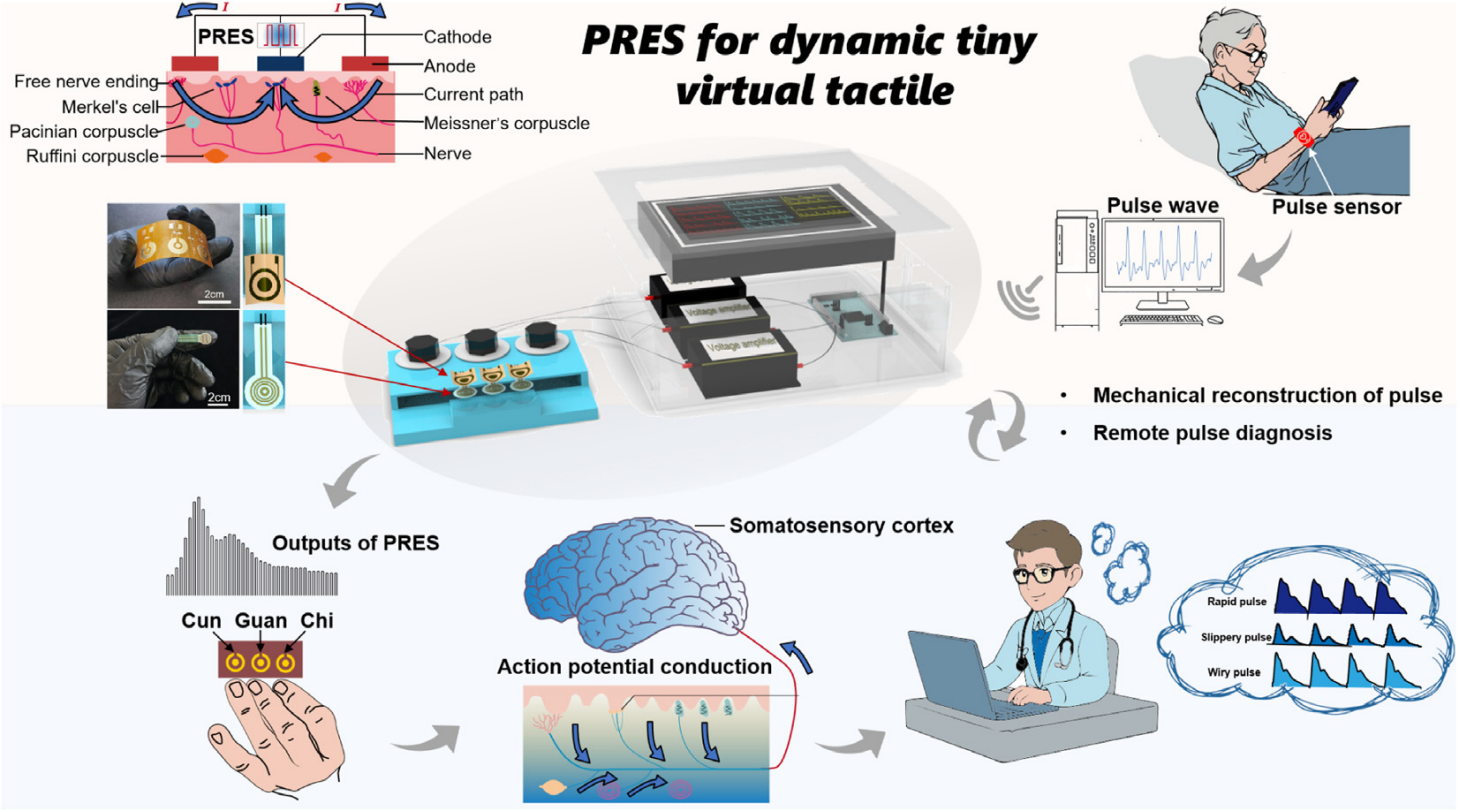
**Schematic illustration of pulse diagnosis and virtual tactile reconstruction using PRES.** The pulse sensor obtains the patient's pulse information and transmits it remotely to the doctor's PRES device. The doctor completes remote pulse diagnosis by touching the electrodes of PRES and sensing the tactile information of the patient's radial artery.

As shown in Fig. 1, we propose a pulse reconstruction electrical stimulator (PRES) for reconstructing the TCM pulse conditions and the user’s wrist pulse according to the signals of wearable sensors, which can generate a dynamic virtual tactile sensation on the fingers to simulate the process of pulse diagnosis realistically. Currently, electrotactile employs vibration and intermittent pressure for various applications, but accurately reconstructing the continuous sense of pressure is a challenging aspect. The PRES can reconstruct the dynamic pressure of various typical pulse conditions and the user’s wrist pulse wave. Three key aspects of our work are encompassed: 1) Dynamic mechanical perception of tiny radial artery pulse through electrical stimulation of fingertips. We have carefully studied the key factors for achieving dynamic haptic sensation through electrical pulse signals, including voltage amplitude, frequency, pulse width, etc. At the same time, we conducted a detailed study on the effect of electrodes on the performance of electrotactile reproduction. 2) Electro-pressure calibration experiment for evaluating the correspondence between electrotactile and haptic force. To better depict the simulation of micro-dynamic pulsation behavior by electrotactile, we designed a control experiment to study the relationship between electrical stimulation parameters and fingertip real mechanical perception. 3) Utilizing the PRES to reconstruct twelve typical TCM pulse conditions. We synthesized pulse condition curves using Gaussian functions and replicated electrical stimulation using PRES. At the same time, we collected the real waveform of the radial artery using a pulse sensor and reconstructed the pulse wave using PRES.

## 2 Materials and methods

### 2.1 Fabrication of PRES and electrodes

The preparation of the PRES included a microcontroller, a voltage amplifier, electrodes, and an electrode mold. The microcontroller was used by Arduino DUE, which was available to output dual-channel 12-bit DACs. The DACs could output the waveform of pulse signals, where the digital range of 0–4095 corresponded to an analog range of 0–3.3 V. The input voltage range of the voltage amplifier was 0–10 V, and the output range was 0–130 V. The maximum bandwidth was 50 kHz. The ESP32 microcontroller and the PPG sensor constitute a pulse acquisition device. The data from the PPG sensor is passed into the ESP32 and transmitted to the ESP8266 via WIFI. Then, the ESP8266 transmits the pulse wave data to the Arduino DUE via the serial port. The PPG sampling rate of ESP32 is 125 Hz.

The flexible printed circuit (FPC) employs a single-layer structure, with polyimide as the substrate material. The FPC was 130 μm in thickness, and the conductive layer was Cu. To accommodate the size of fingers, the anode had an area range of 7–13 mm^2^ while the cathode had an area range of 14–56 mm^2^ (More details can be found in Table S1). A resin mold was designed according to the size of the FPC and printed using a three-dimensional (3D) printer. The design and dimensions of the resin mold are shown in Fig. S3, which effectively restricted the placement position of fingers.

### 2.2 Signal acquisition and processing

The voltage signal of nine pulse conditions was measured by an oscilloscope with a 10 MHz sampling rate. An impedance analyzer was utilized to quantitatively assess the impedance and capacitance between the electrodes and the skin. The measurement model was a parallel combination of resistance (Rp) and capacitance (Cp). With the change of hand humidity, impedance will also change, here humidity and impedance relationship between the experimental design is as follows: environmental humidity of 28% RH, the experimenter finger surface immersed in water, remove the finger (to achieve the state of the water droplets are about to drop), to achieve 100% RH, using micro test 6632 impedance measuring instrument by a fixed time interval on the finger and the electrode to conduct impedance measurements. Place your finger on the electrode and use the MICROTEST 6632 Impedance Meter to measure the impedance of the finger electrode, and remove your finger when the measurement is complete. The time interval from the start of the measurement to the next measurement was 20 s until the finger became dry, and Cp and Rp data were obtained for that time period.

From the experimental results, it can be seen that the values of Cp and Rp at the beginning of the measurement gradually decreased and then stabilized, and then the curve fitting of the data can be obtained as the relationship between Cp and Rp with the change of humidity, with the reduction of humidity Cp shows a slow increase and then decrease the trend of change, Rp shows a slow decrease and then increase the trend of change.

The pulse wave data was measured by the PPG sensor and transmitted to the oscilloscope. The PPG sensor is placed at the Guan of the wrist, which is usually the strongest pulsation point of the radial artery. The Guan is at the position of the radial artery relative to the radial styloid process. When palpating the Guan, one can usually feel the pulse's characteristics, such as its rate, rhythm, strength, and quality. These features can provide valuable insights into a person's health condition. For example, a strong and forceful pulse at the Guan might indicate an excess condition in the body, such as high blood pressure or an acute inflammatory response. On the other hand, a weak and thready pulse could suggest a deficiency, like anemia or a weakened immune system. In traditional Chinese medicine, the Guan is closely related to the spleen and stomach. The spleen is considered the root of postnatal life, responsible for transforming and transporting food and fluids. If the pulse at the Guan is irregular or abnormal, it may reflect issues with the spleen and stomach functions, such as indigestion, bloating, or poor appetite. Moreover, the Guan is also associated with the liver and gallbladder. An abnormal pulse here could indicate emotional stress, liver stagnation, or gallbladder disorders. In addition, the Guan pulse is often used to diagnose diseases related to the digestive system and the liver. For instance, if there is a tight and wiry pulse at the Guan, it might suggest liver qi stagnation, which can lead to symptoms like irritability, chest tightness, and menstrual disorders in women. A slippery pulse at the Guan could indicate the presence of phlegm or dampness in the body, which may cause symptoms like coughing with phlegm or a feeling of heaviness in the limbs. Overall, the Guan pulse is an important diagnostic tool in traditional medicine, providing a window into the body's internal balance and health status.

The formal acquisition process necessitates the utilization of a black wristband to obscure the sensor to avoid being affected by visible light in the environment. Compared with other pulse acquisition sensors, the PPG sensor is an optical sensor with the advantages of non-contact and stable acquisition [34].

The voltage range of the PPG signal is 0 to 3.3 V. Use the Savitzky-Golay filter to smooth the raw signal. The smoothed signal was stored in an array in the microprocessor with a sampling frequency of 125 Hz. The ESP32 reads the signal from the PPG sensor and transmits it to the PRES device via WIFI. The ESP8266 in the PRES device receives the data transmitted by the ESP32 and transfers it to the Arduino DUE microcontroller for storage through serial communication.

The experiments involving human subjects had been performed with the full, informed consent of the volunteer, who was also a co-author of the manuscript. All experiments were approved by the Committee on Ethics of Beihang University (BM20220159).

### 2.3 Synthesis and envelope of pulse condition waveform

The Gaussian model effectively captures the fundamental components of the pulse waveform, encompassing the primary peak, tidal wave, and dicrotic wave. This is attributed to the bell-shaped waveform of the Gaussian function resembling the wave peak of the wrist pulse. Each Gaussian function includes three parameters, where V, T, and U represent the amplitude, peak position, and width of the bell waveform, respectively. As is shown in Eq. 1, the three-term Gaussian can be utilized to describe the percussion wave, tide wave and dicrotic wave in the pulse signal, and its coefficients [V1, V2, V3, T1, T2, T3, U1, U2, U3] are used as the features to represent the amplitude, phase and shape of the pulse waveform.

(1) $$x\left(t\right)=\sum_{i}^{3}V_{i}\exp\left[\frac{-{\left(t-T\right)}^{2}}{U_{i}}\right]$$

The parameters of the Gaussian functions for the four types of pulse conditions reconstructed in this study are shown in Table 2 [35]. After synthesizing the pulse waveforms, 100 electrical pulses are used to envelope the pulse condition waveform. The reason for using 100 pulses to envelope one period is that it can reflect both the dynamic pressure and the temporal characteristics of the pulse wave, including the onset, wave peak, wave trough, and offset. The subject's ability to discriminate continuous pulses is limited, and as the time interval between electrical pulses decreases (that is, the frequency increases), the electrotactile sensation also changes from discrete to continuous.

**Table 2:** **Parameters of the Gaussian function of four pulse conditions**.

Pulse condition	V1	V2	V3	T1	T2	T3	U1	U2	U3
Normal pulse	40	20	5	10	20	35	16	49	196
Slippery pulse	39	11.7	2.1	10.2	21.8	35.2	15.2	82.8	289
Wiry pulse	23.2	20.1	14.3	15.1	24.9	35.1	16.8	104.8	72.3
Rapid pulse	23.8	20.2	10	9.8	14.9	35.1	12.2	99.4	246.5

## 3 Results

### 3.1 Design of the PRES and electrodes

**Fig. 2:**
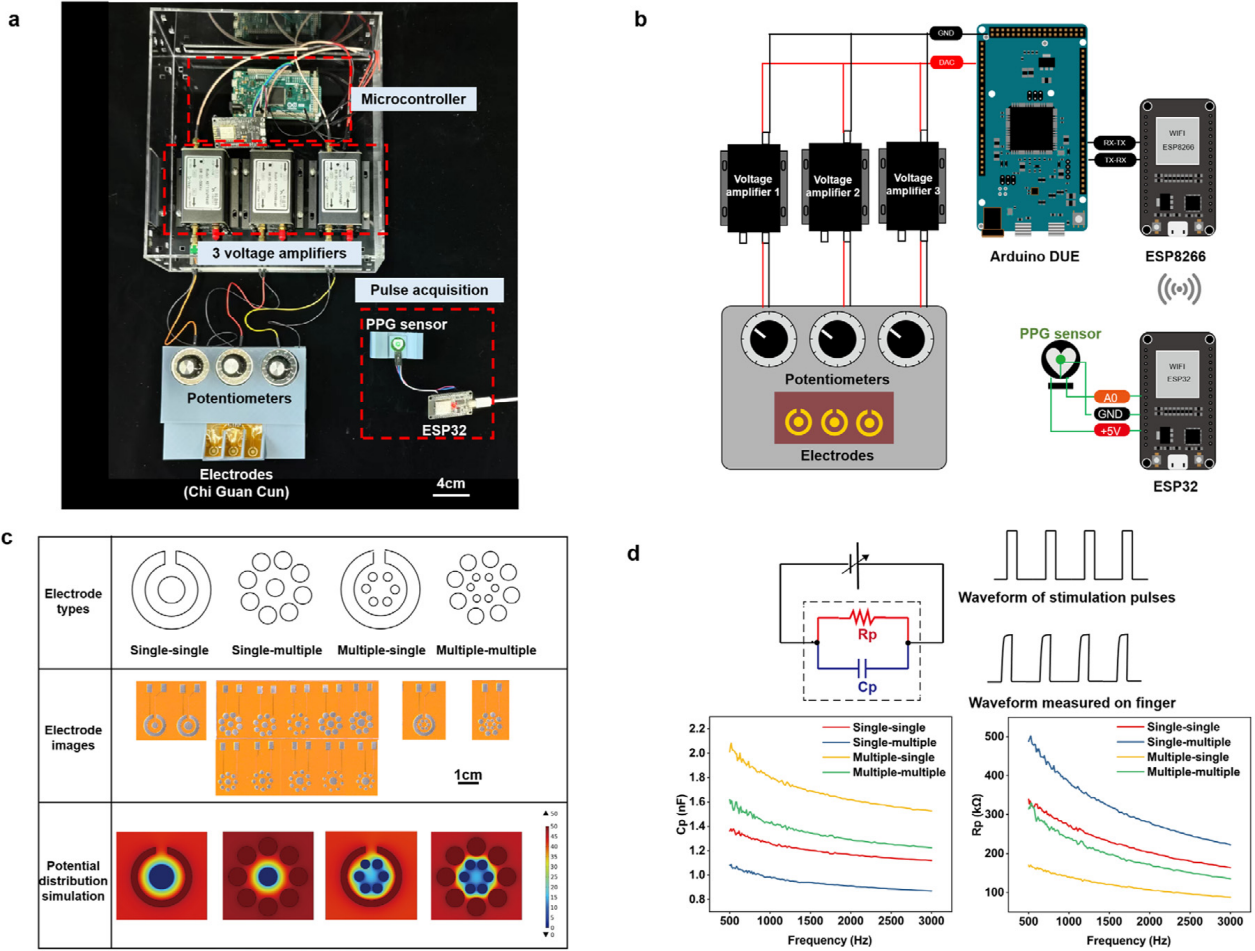
**Electrode designs and results of simulation and impedance analysis.** (a) Physical map of PRES. (b) Circuit diagram of PRES. (c) Images of electrodes with different shapes and sizes and COMSOL simulation results. The design diagram of the electrode circuits is shown in Fig. S4. Finite element simulation by COMSOL to present the potential distribution for four electrode designs. (d) Impedance model of the equivalent circuit and impedance results of four electrode designs. Due to the capacitive effect of the skin, the shape of square waves received by the finger will distorts.

**Fig. 3:**
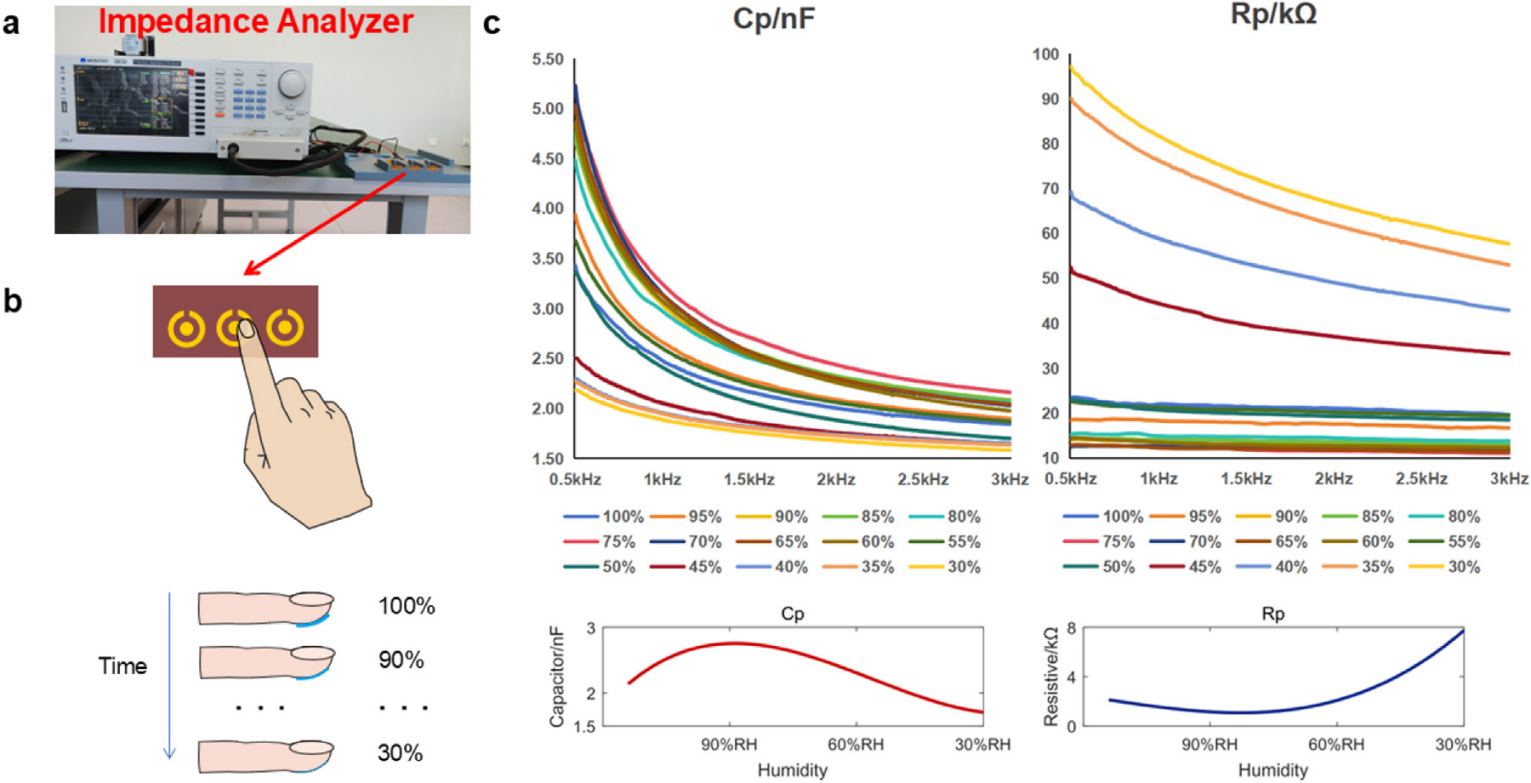
**Impedance and humidity change experiment.** (a) Experimental scenarios with the MICROTEST 6632 impedance analyzer. (b) Measurement methods and experimental procedures. (c) Cp, Rp versus humidity curves with performed data fitting.

As shown in Fig. 2, Fig. 3 and b, the tactile reconstruction system includes two components: the PRES and electrodes. The function of PRES is to apply pulse voltage signals to electrodes. Firstly, the waveform data of the pulse conditions is stored in the microcontroller. The digital-to-analog converter (DAC) module of the microcontroller generates pulse voltage signals, which necessitate transmission to voltage amplifiers due to the high impedance of the finger corneum. Each electrode is connected to a potentiometer with a resistance of 100 kΩ, allowing for convenient control of the stimulation intensity through voltage division. The range of stimulator parameters used in the subsequent experiment is shown in Table S2. According to the preliminary experiment, a short-lived sensation of pressure can be felt at the fingertip when applying a 20–100 V voltage pulse signal (with a pulse width ranging from 20 μs to 2 ms). Parameters such as pulse width, frequency, and output amplitude can be individually adjusted to meet the requirements of this experimental study. The demonstration of the threshold testing process is shown in Movie S1.

Researchers use the activating function (AF) to explain the principle of how to utilize selective stimulation to generate pressure. AF is as shown in Eq. 2:

(2) $$\text{AF}=\frac{\partial^{2}\psi }{\partial r^{2}}$$

where potential distribution Ψ is applied to the surface of a subcutaneous axon, and r is the distance along a nerve axon. The activating function of the horizontal axon is positive when the surrounding anode is connected to the positive electrode of the stimulator and the central cathode is connected to the negative. Therefore, the activation of nerve axons connected to Merkel cells results in the generation of pressure sensation. This mode of selectively stimulating to create pressure is called the SAI mode. And the increasing number of surrounding anodes can result in a superposition of AF values, allowing for more precise control of the position of pressure below the cathode [26].

In TCM, doctors take the pulse at three positions on the wrist known as Cun, Guan, and Chi. Therefore, three separate electrodes are designed to represent different positions of the radial artery in the wrist. Different electrode designs influence the distribution and intensity of sensations [36]. Therefore, we devised four electrode types and parameters as presented in Fig. 2c and Table S1. Electrodes contain peripheral anodes and cathodes, which are respectively designed in different shapes (single-single, single-multiple, multiple-single, multiple, and multiple) and sizes. Simulation of potential distribution is calculated by COMSOL (Fig. 2c). It illustrates the instantaneous distribution of electric potential between the electrode and the skin when a voltage of 50 V is applied [37,38]. Moreover, an impedance analyzer was used to measure the equivalent capacitance (Cp) and resistance (Rp) of fingers and electrodes (Fig. 2d). We applied the same electrical stimulation to each electrode type; the single-single electrode (anode area is 13 mm^2^) exhibited the most comfortable sensation and required the lowest voltage for the same sensation intensity. Hence, this electrode will be employed for subsequent electrical stimulation applications.

### 3.2 Tactile intensity grading and electro-pressure calibration

**Fig. 4:**
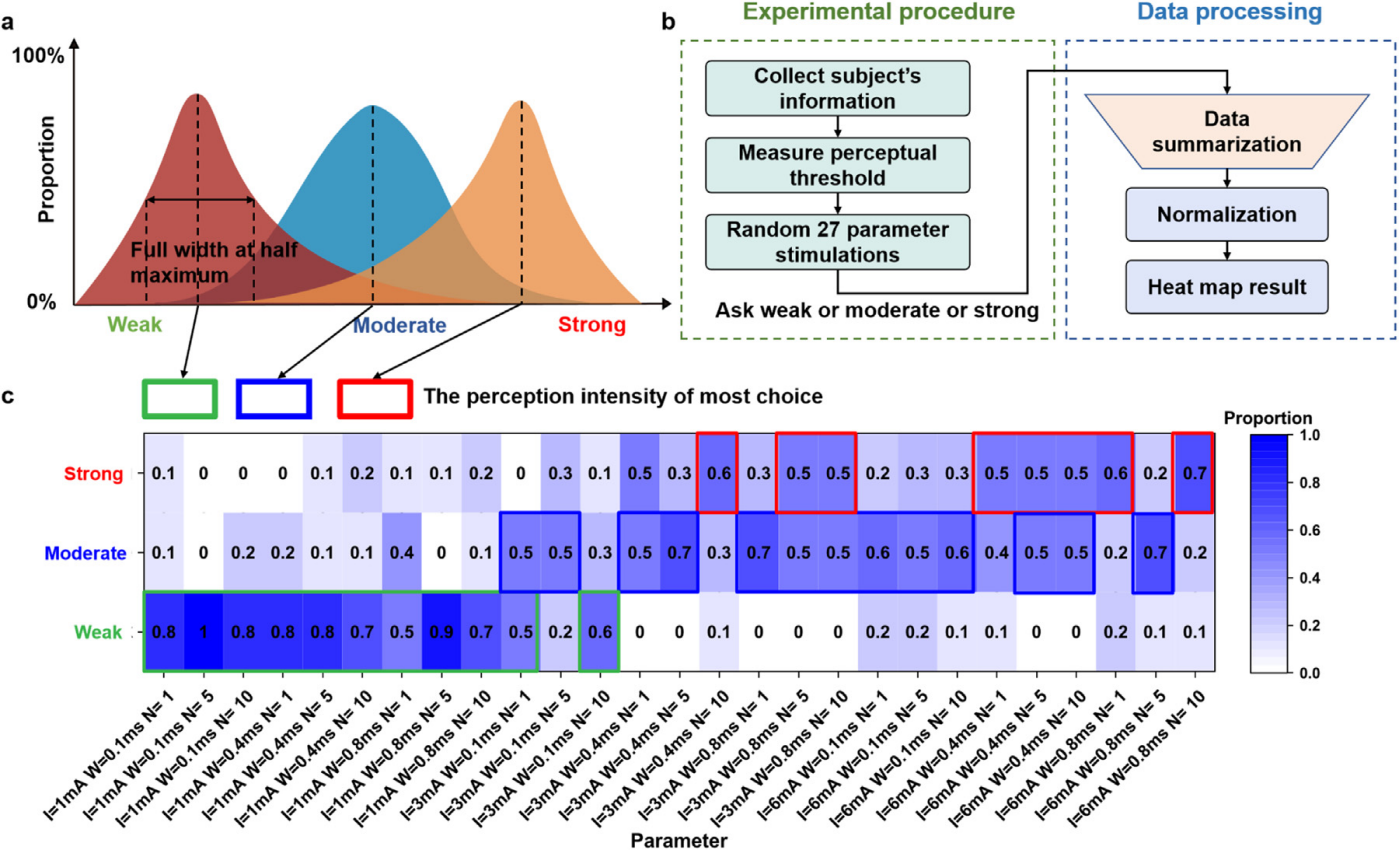
**Schematic diagram and results of tactile intensity grading experiment.** (a) Each set of parameters may have different choices due to human subjectivity. Full width at half maximum indicates the concentration range and symmetry of perceived intensity. The option exhibiting the greatest proportion of each parameter set was marked by rectangle. (b) Flow diagram of tactile intensity grading experiment. (c) Heatmap of subjects' perception of electrical stimulation intensity. The number and color depth represent the proportion of the perceived intensity to the overall "weak, moderate, and strong." The rectangular box represents the highest proportion of subjects choosing under this parameter. When current is 1 mA, it is preferable to choose a weak intensity. When current is 3 mA, it is preferable to choose a moderate intensity. When current is 6 mA, it is preferable to choose a strong intensity.

The pressure pulsation at the radial artery exhibits dynamic variations, necessitating diverse levels of perception intensity through adjustments in electrical stimulation parameters. These parameters encompass current pulse amplitude (I), pulse width (w), and pulse number (N), all of which are associated with perceived intensity [[39], [40], [41]]. There are formula relationships between the three parameters when maintaining consistent perceptual intensity. Eqs. 3 and 4 are shown below:

(3) logw=a+b*logN

(4) $$I*w^{-0.5}=10^{c}$$

where a, b, and c are constant [42,43]. There exists a significant linear relationship between logW and logN. Eq. 3 indicates that pulse width and frequency behave as logarithmic functions. Eq. 4 indicates that under the same perceived intensity, a shorter pulse width typically requires a higher current intensity. As depicted in Fig. 4a, due to subjectivity, individuals may exhibit varying tactile intensities towards distinct parameters. Employing psychophysical methods, we conducted the tactile intensity grading experiment to explore the correlation between electrical stimulation parameters and perceived intensity. Subsequently, based on the aforementioned stimulation parameters, subjects’ corresponding perceptions of intensity were recorded with 27 combinations of *I* = 1, 3, 6 mA, *w* = 100, 400, 800 μs, and *N* = 1, 5, 10. The range of electrical stimulation parameters selected ensures that the subjects are safe and comfortable during the electrical stimulation experiments. The entire duration of the experiment did not cause fatigue or pain from electrical stimulation. The experimental flow chart is shown in Fig. 4b The specific steps of the experiment are as follows:

(1) Collect the subject’s information (e.g., gender, age, and strong hand) and provide explanations on device usage and experimental safety.
(2) Due to individual variations, it is imperative to assess the subjects’ perceptual threshold (the current amplitude at which they begin experiencing pressure). Elucidate and explore three levels of perceptual intensity—weak, moderate, and strong—while repeatedly training to enhance the subject's memory.
(3) The subject is randomly assigned a stimulus parameter and stimulated periodically five times, allowing 10 s for the subject to report the perceived pressure intensity (weak, moderate, or strong). This process is repeated for all 27 combinations of parameters. The statistical table utilized in the experiment is presented in Table S3.

Following the experiment, we collected the perceptual intensity choices made by all subjects. Ultimately, we can ascertain the frequency of selecting “weak, moderate, and strong” for each parameter. The experimental results can serve as a valuable reference for determining the parameters utilized in the pulse condition waveform envelope.

**Fig. 5:**
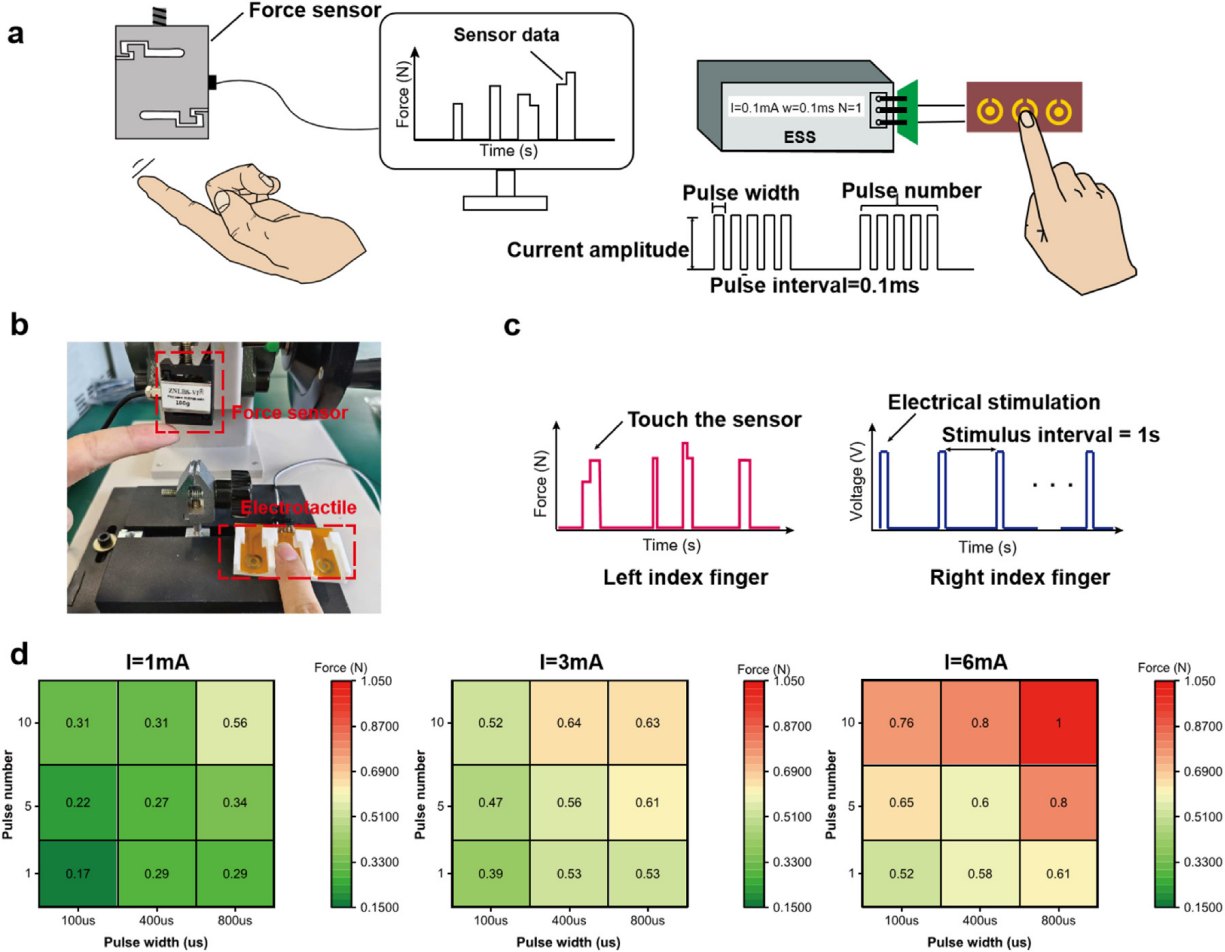
**Electro-pressure calibration experiment for 27 electrical parameter combinations and pressure calibration result.** (a) Schematic diagram of a force calibration experiment. (b) Physical map of experimental environment. (c) Comparison of results of pressure sensor output signal and PRES input signal. (d) Heat map of electro-pressure calibration. The magnitude of force at different pulse widths and pulse numbers when *I* = 1 mA, *I* = 3 mA, and *I* = 6 mA res-pectively.

The statistical results of the intensity grading experiment are shown in Table S4. A heatmap depicting the proportion of 27 parameters is illustrated in Fig. 4c. The higher the proportion of selection under this parameter condition, the deeper the blue color. The option exhibiting the greatest proportion of each parameter set was marked. The results indicate that the intensity of pressure can be controlled by adjusting the parameters of PRES. The statistical result of the electro-pressure calibration is shown in Table S5. A heatmap depicting the accuracy rates of 27 parameter sets is illustrated in Fig. 5. The output pressure of the PRES ranges from 0.17–1 N. Generally, an increase in pulse width, pulse number, and pulse amplitude results in a corresponding augmentation of pressure presentation. In addition, the increase in stimulation intensity caused by pulse number is greater than that of pulse width.

### 3.3 Reconstruction of pulse conditions and waveforms

The tactile reproduction of the wrist pulse is based on the pulse wave waveform, and PRES can reproduce any pulse wave waveform. Therefore, waveform data acquisition is one of the main contents of pulse reconstruction and determines the quality and accuracy of reconstruction. In this article, pulse waveforms are categorized into pulse condition waveforms and actual measured pulse waveforms, corresponding to pulse condition training and remote pulse diagnosis applications, respectively. The pulse condition waveforms are defined based on mathematical models and relevant literature, while the actual measured pulse waveform is obtained through PPG sensors.

**Fig. 6:**
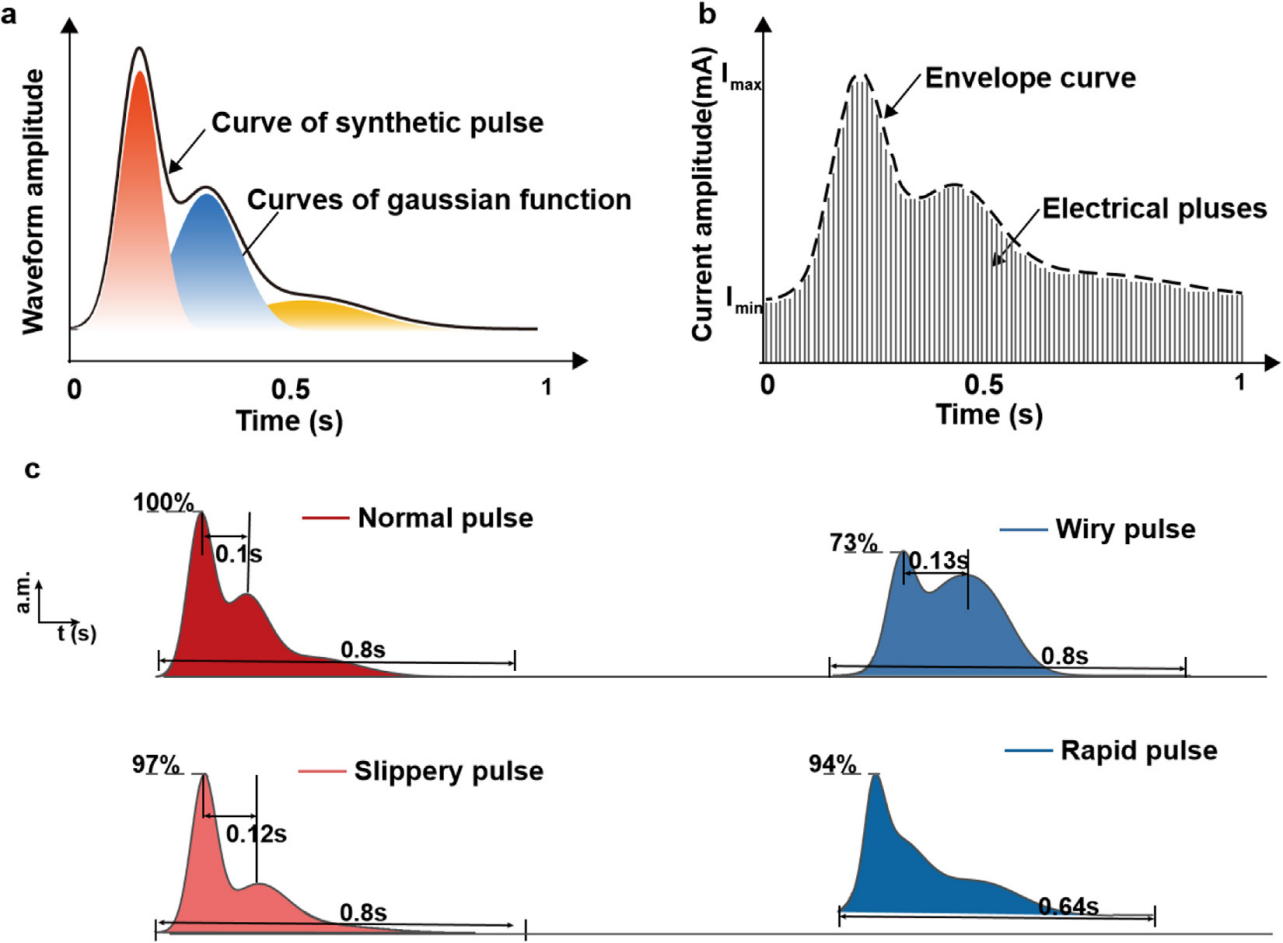
**Synthetic and envelop of pulse conditions.** (a) Synthesized pulse condition waveform (normal pulse) using three Gaussian functions. (b) Envelop curve of pulse condition with different pulse amplitudes and time sequences. There are using 40 electric pulses to envelop the curve for better representation. (c) Four pulse condition curves by adjusting the parameters of Gaussian function.

The differences in pulse conditions can be reflected in the differences in waveforms, such as time domain, frequency domain, and time-frequency domain characteristics. Mathematical models are adopted to fit the wrist pulse signal, such as the Gaussian model [44], discrete Fourier series model [45], and autoregressive (AR) model [46]. The Gaussian model is a synthesis of pulse waveforms utilizing three adjustable parameters derived from Gaussian functions, which effectively capture the fundamental time-domain characteristics of pulse waves. As shown in Fig. 6, we synthesized four standard pulse condition waveforms using Gaussian functions and used electric pulses to envelope the waveforms (detailed formulas and data in methods). In addition to synthesizing four pulse waveforms, we have successfully replicated a total of twelve pulse conditions. Among them, three pulse conditions (floating pulse, deep pulse, and hidden pulse) are achieved through pressure feedback by a force sensor, while the remaining nine pulse conditions are achieved by adjusting the period and rhythm of the standard waveform.

**Fig. 7:**
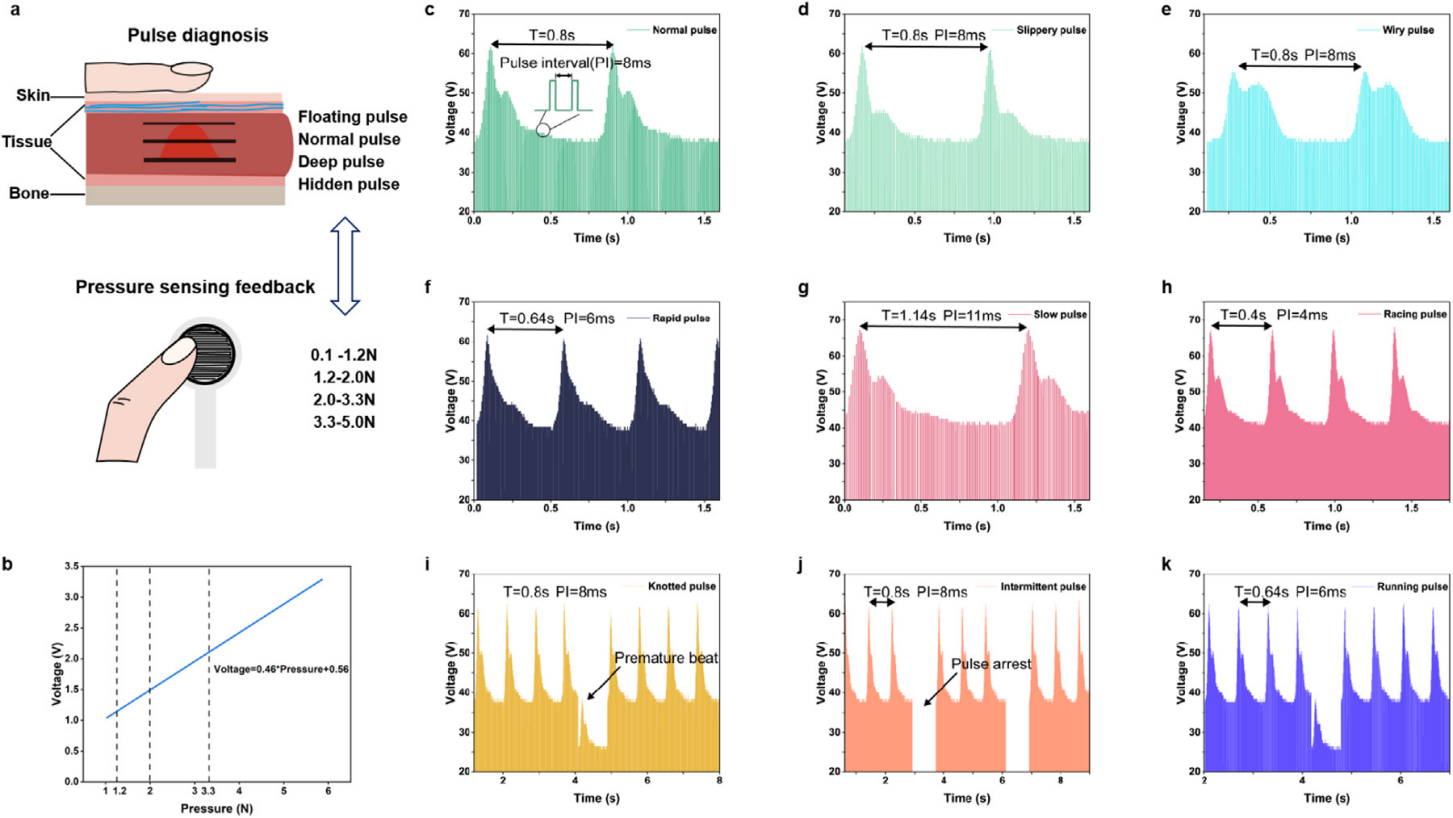
**Voltage signal of twelve pulse conditions generated by the PRES.** (a) The finger force is quantified by employing a piezoresistive pressure sensor to facilitate electrotactile feedback. Four pulse conditions correspond to different pressure ranges. (b) The linear response of force sensor for pressure measurement. (c–k) The voltage signals of the nine pulse conditions output by PRES. The envelope of each pulse period consists of 100 electrical pulses. The periods of normal pulse, slippery pulse, and wiry pulse are 0.8 s. The periods of slow pulse, delayed pulse, and rapid pulse are 0.64 s, 1.14 s, and 0.4 s respectively by adjusting the time interval between pulses.

In TCM, the pressure of the finger applied to the radial artery by doctors is crucial, as different levels of finger pressure yield varying tactile feedback. Moreover, different physicians have varying habits when it comes to the pressure applied with their fingers, including the speed of pressing, the sequence of pressing at the Cun, Guan, and Chi positions, as well as the pressure used. Therefore, it is necessary to use three pressure sensors to measure the finger force in real time. According to the pressure of the finger required for perceiving the optimal pulse, pulse conditions can be classified into floating pulse, normal pulse, deep pulse, and hidden pulse. As shown in Fig. 7a, the required finger force range for a floating pulse, normal pulse, deep pulse and hidden pulse gradually increases, respectively from 0.1–1.2 N, 1.2–2 N, 2.0–3.3 N and 3.3–5 N, indicating that only within this pressure range will PRES output an electrical signal. The pressure is measured by a piezoresistive pressure sensor, which has a suitable range. The sensor is connected to a linear circuit module, which can provide a linear output voltage signal for the ADC module of the microcontroller to read. The linear voltage response of the piezoresistive pressure sensor is shown in Fig. 7b.The microcontroller calculates the force value based on the voltage and determines whether to output an electrical signal. For convenience, the waveforms for the floating pulse, deep pulse, and resting pulse are the same as the normal pulse.

The pulse condition reconstruction voltage signals generated by PRES are depicted in Fig. 7c–k. The normal pulse is a characteristic pulse condition observed in healthy individuals, typically exhibiting three peaks with a period duration of 0.8 s, with an interval between pulses of 8 ms. The slippery pulse exhibits two peaks with steeper ascending and descending slopes. Additionally, a distinct dicrotic wave can be observed. The pressure waveform of the slippery pulse exhibits characteristics reminiscent of the rolling motion of beads. The wiry pulse exhibits a wide and high-amplitude main wave, accompanied by an earlier and larger amplitude dicrotic wave. This results in a continuous pressure sensation on the fingers, indicating the tactile characteristics of the touch string. The waveform of the rapid pulse is similar to that of a normal pulse, indicating a shorter cardiac period, which can be achieved by reducing the time interval between pulses to 6 ms. The period of the rapid pulse is 0.64 s and is adjustable. Similarly, the racing pulse and slow pulse can also be adjusted by the time intervals of 4 ms and 11 ms, respectively. The intermittent pulse exhibits periods of irregularity, brief pauses and interruptions, as well as slow and halted beats. In contrast, the knotted pulse demonstrates regular pauses with longer resting intervals. In addition, knotted and intermittent pulses are related to bradyarrhythmia. Therefore, we employ a premature beat and pulse arrest as indicators of the knotted pulse and intermittent pulse, respectively. The running pulse exhibits a shorter pulsation period in comparison to the knotted pulse. The human-computer interaction interface of pulse condition reconstruction is shown in Movie S2.

**Fig. 8:**
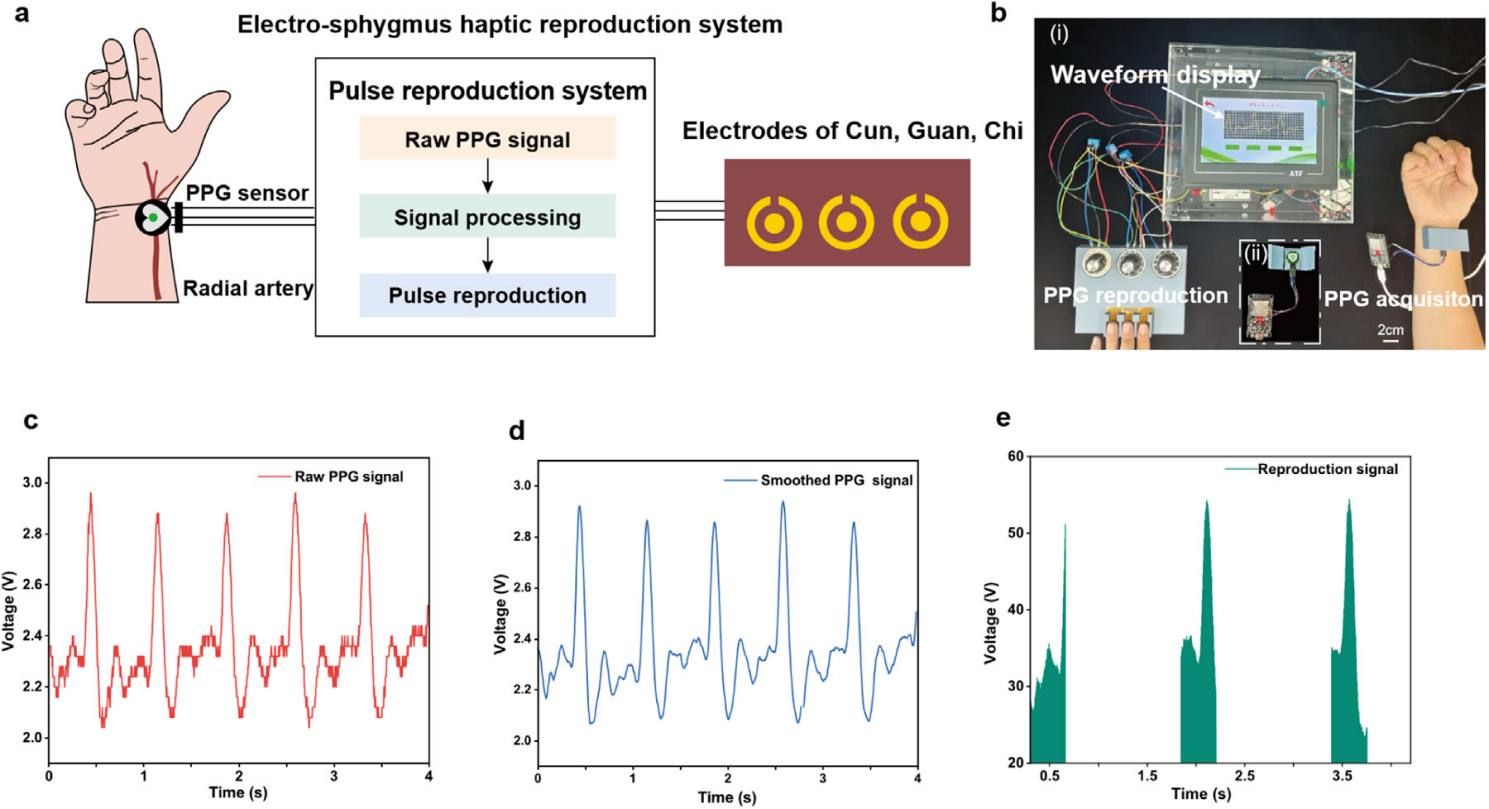
**Results of user’s PPG haptic reconstruction.** (a) Process of PPG signal acquisition and PPG tactile reconstruction. (b) (i) Experimental scene picture for the measurement of PPG signals and tactile reproduction. (ii) The picture of the PPG acquisition device, including the PPG sensor and ESP32 microcontroller. (c) Raw PPG curve collected by PPG sensors. (d) Smoothed PPG curve. (e) The PRES output of radial artery waveform.

The PRES is capable of replicating any waveform, and we have acquired and reconstructed a waveform of the user's radial artery. As shown in Fig. 8a and b, the pulse wave of the radial artery of the subjects was acquired using a PPG sensor. The methodology for assessing pulse waves using PPG is elucidated in the supporting information. The measured PPG signal is transmitted to PRES via the WIFI of ESP32. The screen of PRES can display the measured PPG waveform. Apply a Savitzky-Golay filter to smooth the sensor data and employ the PRES for haptic reconstruction. The advantage of this filtering algorithm lies in its ability to effectively eliminate noise while preserving the inherent shape of the signal. After the PPG waveform data is smoothed, it is then transmitted to the device for reconstruction. Fig. 8c–e illustrates the curves of the raw and smoothed signal and reconstruction outcomes of the PPG signal. The SNR of the signal before and after smoothing is calculated here: the SNR of the signal before smoothing is 19.94 dB, and the SNR of the signal after smoothing is 19.88 dB The SNRs before and after smoothing are the same or very close to each other, which indicates that the smoothing operation has minimal effect on the fidelity of the signal. Similarly, this method can replicate the tactile sensation experienced at the radial artery of any user, thereby facilitating its application in remote pulse diagnosis. The above process demonstrates the application of remote pulse diagnosis in this study, which wirelessly transmits the user's radial artery waveform to the doctor’s PRES device for haptic reproduction.

## 4 Discussion

In conclusion, we report a pulse reconstruction electrical stimulator (PRES) based on electrotactile, which presents a potential application for rendering dynamic pressure sensation for pulse diagnosis. The users can feel the dynamic pressure of different pulse conditions and their wrist pulse by putting their fingers on the electrodes of the PRES. Experimental results on subjects indicate that the perception intensity of electrotactile and the features of pulse condition can be controlled by adjusting parameters such as voltage amplitude, pulse width, number of pulses, and the pulse interval.

We studied the relationship between electrical parameters and tactile intensity through mechanical evaluation and blind testing methods, and optimized electrode parameters, electrical stimulation waveforms, and device interaction interfaces based on this. The subject experiment verified that the PRES can reconstruct the different intensities of the pressure of pulse conditions, which reflected the applicability and practicability of the pulse reconstruction device designed for pulse diagnosis training.

Compared with previous pulse reconstruction devices, the proposed tactile-based pulse diagnosis reconstruction device in this paper exhibits notable characteristics, including compact size, high resolution, and adjustable parameters. It effectively presents intricate waveform information of the pulse wave. Our device integrates force sensors for feedback on finger force, allowing the output electrical signal of PRES to be controlled based on the user's finger force. The electrotactile device has been effectively validated in simulating twelve distinct pulse conditions. Twelve pulse conditions based on waveform differences are synthesized. The device described in this paper can reconstruct any pulse waveform. Moreover, our device can realize remote pulse diagnosis, collect pulses using a pulse sensor, and transmit pulses wirelessly to PRES for reconstruction.

The results of the subject experiment demonstrate that, through varying combinations of electrical stimulation parameters, the subjects exhibit a high recognition rate in distinguishing levels of pressure sensation intensity and detecting changes in intensity. In previous studies, mechanical stimulation methods have been predominantly employed in existing devices for reconstructing pulse waveforms. Compared with them, the proposed electrotactile pulse condition reconstruction device has a rapid response, abundant adjustable parameters, and can accurately capture the time-domain features of pulse condition waves. Moreover, it offers enhanced portability and cost-effectiveness in comparison to commercial training equipment, thereby enabling the potential for training in pulse palpation or remote pulse diagnosis [47].

In the future, we plan to explore the possibility of simulating more types of pulse conditions with force sensors, electrode designs, and collecting more pulse condition curves. The research on reconstructing pulse conditions focuses on common types, including the normal pulse, slippery pulse, and rapid pulse. In the future, we plan to collaborate with hospitals and professional physicians to collect additional pulse conditions. We will conduct double-blind experiments and establish a comprehensive database of pulse conditions. It is anticipated that the size of the equipment will be further reduced in the future, for instance, by designing even smaller voltage amplifiers and microcontrollers. Furthermore, we will integrate a wearable pulse acquisition device and pulse condition recognition to attain long-term acquisition of wrist pulse signals and achieve real-time remote diagnosis.

## CRediT authorship contribution statement

**Xiaowei Zhao:** Writing – original draft, Methodology, Investigation. **Yuxin Song:** Writing – original draft, Methodology, Investigation. **Bojing Shi:** Writing – review & editing, Writing – original draft, Supervision, Project administration, Methodology, Investigation, Funding acquisition, Conceptualization. **Yubo Fan:** Writing – review & editing, Visualization, Supervision, Project administration, Methodology, Funding acquisition, Conceptualization.

## Appendix Supplementary materials

Image, application 1

Image, video 2

Image, video 3
