# Supplementary material for: Continuous dynamic microforce reconstruction using electrical stimulation for remote pulse diagnosis
Source: Fundam Res. 2025 Sep 9;5(6):2453–62. doi: 10.1016/j.fmre.2025.09.002 (PMC12744653; doi:10.1016/j.fmre.2025.09.002)
Supplement: Supplementary file 1 [file mmc1.docx]

Supporting Information

**Continuous dynamic micro force reconstruction using electrical stimulation for remote pulse diagnosis**

*Xiaowei Zhao*,*^#^* *Yuxin Song,^#^* *Bojing Shi,* and Yubo Fan**

**Affiliation**: Key Laboratory for Biomechanics and Mechanobiology of Ministry of Education, Beijing Advanced Innovation Center for Biomedical Engineering, School of Biological Science and Medical Engineering, School of Engineering Medicine, Beihang University, Beijing 100191, China.

#These authors contributed to the work equally and should be regarded as co-first authors.
**Corresponding author**: E-mail: bjshi@buaa.edu.cn (B.S.), yubofan@buaa.edu.cn (Y.F.)

**Pulse diagnosis and pulse conditions**

Traditional Chinese medicine (TCM) pulse diagnosis assesses diseases by palpating patients’ pulses in the wrist artery using fingertips. The doctor places the index finger, middle finger, and ring finger on the patient's wrist, with their positions corresponding to Cun, Guan, and Chi, respectively. The positions of Cun, Guan, and Chi are illustrated in Fig.S1. The doctor feels the pulse by constantly adjusting the pressure on the patient’s wrist with their fingertips. Then, the doctor gets the information about position, strength, trend, tensity, shape, width, and variation of the rhythm of the radial artery. Distinct pulse conditions observed in the Cun, Guan, and Chi positions of both left and right hands are indicative of varying physiological conditions within the body.

According to the widely accepted pulse classification method, individuals’ pulses are categorized into normal pulse and 27 additional pulse conditions. These 27 pulse conditions are categorized based on their forms, durations, locations, intensities, tendencies, rhythmic fluctuations, and other characteristics. The objectification of pulse diagnosis facilitates students to understand and master the theory of pulse patterns. By acquiring and analyzing wrist pulse wave signals and reconstructing the tactile sensation of pulse waves, we can promote applications for pulse diagnosis training and remote diagnosis. Researchers have conducted extensive studies on the objectification of pulse conditions, including pulse acquisition, feature extraction, and curve fitting.

**Electrotactile theory**

Electrotactile is a haptic that utilizes electrical current through a pair of electrodes (anode and cathode) placed on the skin surface to directly stimulate sensory nerves and mechanoreceptors in the skin. And tactile sensation is formed in the brain after the skin nerves receive the electrical current. Electrotactile stimulators possess the ability to exhibit enhanced resolution and quicker responses, all while maintaining a lightweight and flexible design. Its uses encompass assistive devices for individuals with visual impairments, immersive experiences in virtual reality and telepresence, as well as touch-sensitive panels that provide tactile feedback.

The structure of the skin can be divided into three layers: epidermis, dermis, and hypodermis. As shown in Supplementary Fig.2, there exist four distinct categories of mechanoreceptors found within the human dermis, namely Meissner corpuscle (RA), Merkel cell (SAI), Ruffini ending (SAII), and Pacinian corpuscle (PC). Mechanoreceptors are capable of converting the energy generated by external stimuli on the human body into neural impulses, resulting in action potentials in nerve fibers. Action potentials are transmitted through the nervous system to the brain and generate tactile consciousness in the somatosensory cortex. Electrotactile mainly stimulates the superficial layer mechanoreceptors, such as Merkel’s discs and Meissner’s corpuscles.

By adjusting the parameters such as pulse amplitude, frequency, and pulse width of the stimulating voltage or current, it is possible to provide the stimulated area of human skin with a discernible sense of pressure, vibration, and texture. Electrical stimulation parameters include polarity, pulse width, pulse amplitude, pulse frequency, and pulse number. These parameters have an impact on the comfort, dynamic range, threshold, and perceived intensity of electrotactile sensation. Pulse polarity includes positive, negative, and biphasic. Compared with single-phase stimulation, biphasic stimulation with alternating positive and negative charges may have advantages in eliciting various tactile sensations, because biphasic stimulation can reduce the polarization caused by the first stimulation, increasing the comfort of electro-tactile. The square wave is commonly employed as the waveform in electrotactile perception, with pulse amplitude, pulse width, and pulse frequency serving as the primary parameters of this waveform. The perceived intensity of electrotactile is directly influenced by the amplitude and width of the pulse. The perception of electro-tactile sensation, such as vibration and tingling, is primarily regulated by the frequency of the electrical pulse.

**Photoplethysmography (PPG)**

The PPG waveform is primarily affected by the rhythmic variation of blood flow through a tissue site, which can be generated either by the transmission of light or its reflection from body tissues such as skin, bone, blood, and arterial vessels. And the PPG waveform can be measured at the fingertips, the radial artery, and the carotid artery. In clinical settings, PPG has been utilized for monitoring physiological parameters such as blood oxygen saturation, heart rate, respiration, and blood pressure. The fundamental process involves an assembly comprising an infrared source and a detector, where the light emitted by the source traverses through tissues and cells before reaching the detector. The extent to which light is absorbed depends on the blood volume flow during each cycle of cardiac activity.


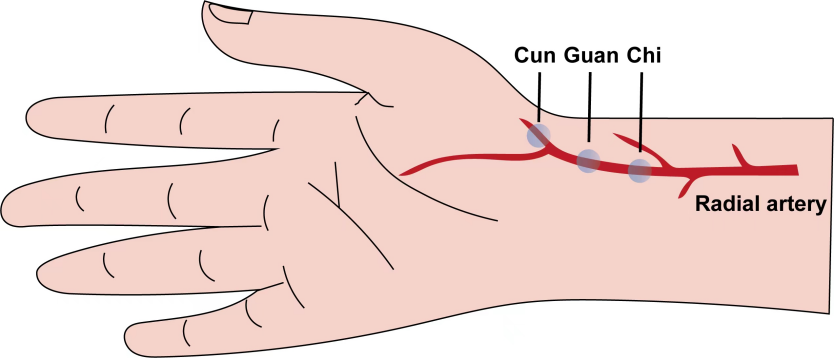


**Fig.S1**. Schematic diagram of the position of Cun, Guan, and Chi.

Pulse diagnosis, a fundamental diagnostic technique in Traditional Chinese Medicine (TCM), involves analyzing the condition of a patient's pulse at specific locations on the wrist to assess their health status and underlying imbalances. The three main locations, known as "Cun," "Guan," and "Chi," correspond to different organs and bodily functions, offering valuable insights into the individual's overall well-being.

Cun: Located nearest the wrist bone, the Cun position represents the superficial and uppermost aspects of the body, corresponding to the Heart and Lungs in TCM theory. The quality, strength, and rhythm of the pulse felt here can reveal conditions related to the circulation of blood, Qi (vital energy), and respiration. For example, a weak or thready pulse at Cun may indicate deficiencies in the Heart or Lungs, while a rapid or forceful pulse could suggest heat or excess conditions.

Guan: The Guan position lies in the middle of the wrist, representing the middle aspects of the body and associated with the Spleen, Stomach, and Liver. This area provides information about digestion, nutrient absorption, and emotional states. A full and steady pulse at Guan suggests good functioning of these organs, while an irregular or congested pulse may point to imbalances such as digestive disturbances or emotional turmoil.

Chi: The furthest from the wrist bone, the Chi position represents the lowermost aspects of the body, associated with the Kidneys and the Urinary Bladder. The pulse here indicates the strength of one's foundational constitution, reproductive health, and water metabolism. A deep, slow, and strong pulse at Chi signifies a strong foundation and good Kidney function. Conversely, a weak or excessive pulse at this location could suggest deficiencies in Kidney Qi, affecting energy levels, sexual function, or fluid balance.

Together, the assessment of the Cun, Guan, and Chi positions forms the basis of pulse diagnosis in TCM, allowing practitioners to gain a comprehensive understanding of the patient's health status and develop targeted treatment plans to restore balance and harmony within the body.


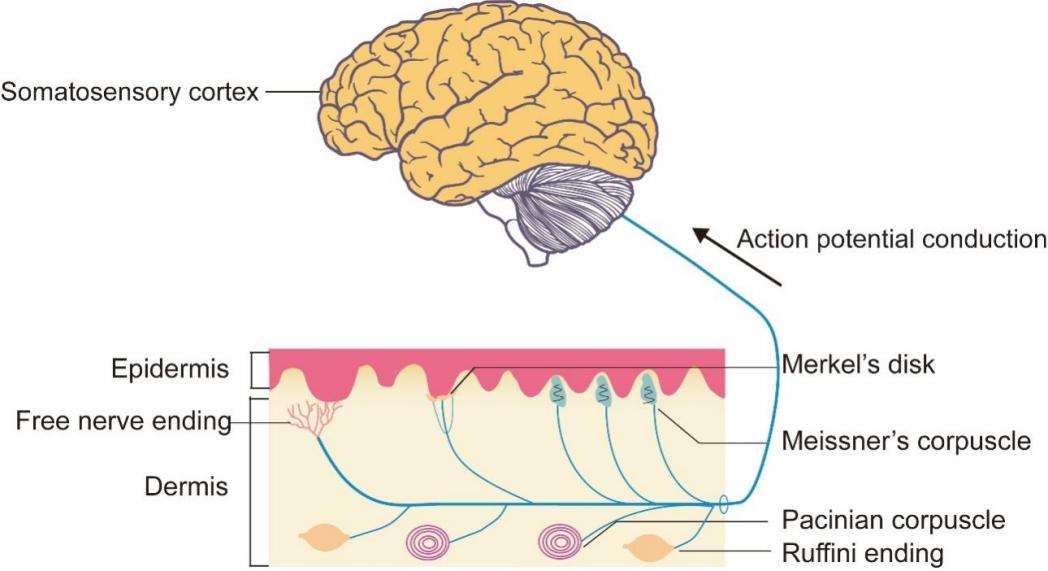


**Fig.S2.** Schematic of the neural tactile sensing system.

The fingertips are remarkable for their sensitivity, thanks to a dense network of sensory receptors embedded within their skin. These receptors, which include Meissner’s corpuscles, Merkel’s discs, and Pacinian corpuscles, enable us to perceive an array of tactile sensations with exquisite precision.

Meissner’s corpuscles, abundant in the superficial layers of the skin, specialize in detecting fine, two-point discrimination and vibratory stimuli. They contribute to our ability to differentiate between textures, recognize details in objects we touch, and even sense the smallest vibrations.

Merkel’s discs, located deeper in the dermis, are responsible for providing tactile information about continuous pressure and low-frequency vibrations. They enhance our sense of touch's ability to distinguish between firmness and softness, contributing to our overall understanding of the physical properties of objects we encounter.

Pacinian corpuscles, or Raphe-spiral corpuscles, are found deeper still and are particularly sensitive to rapid, high-frequency vibrations. They play a key role in recognizing the rapid changes in pressure and vibration patterns associated with, for instance, the texture of rough surfaces or the subtle movements of our fingertips as we type on a keyboard.

These diverse receptors work in harmony to transform mechanical stimuli into neural signals that are relayed to the brain, where they are interpreted as our perception of touch. The fingertips’ rich sensory endowment is a testament to the importance of touch in our daily lives, allowing us to interact with our environment, manipulate objects, and form emotional connections with others.


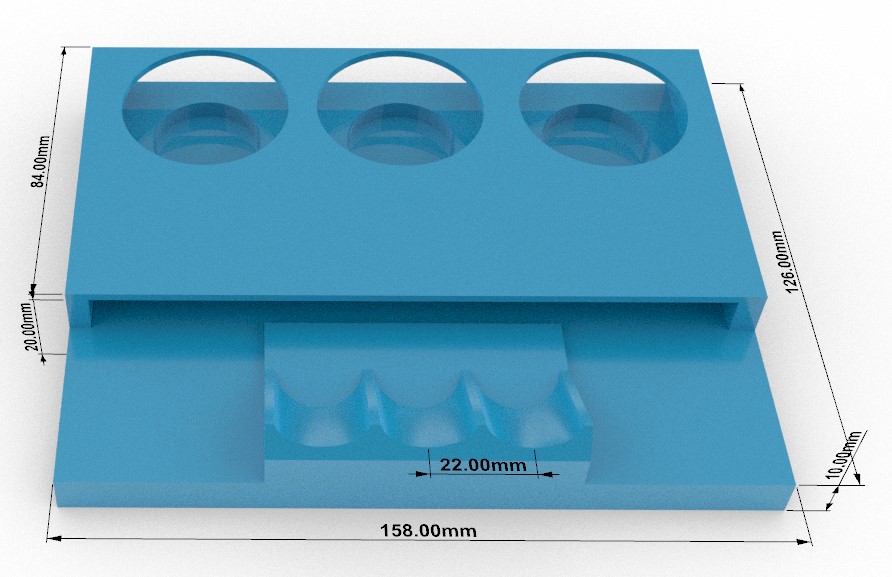


**Fig.S3.** Drawing and dimension marking of the mold. Three circular areas hold potentiometers. The size of the flexible electrode mold is designed according to the size of the fingers.


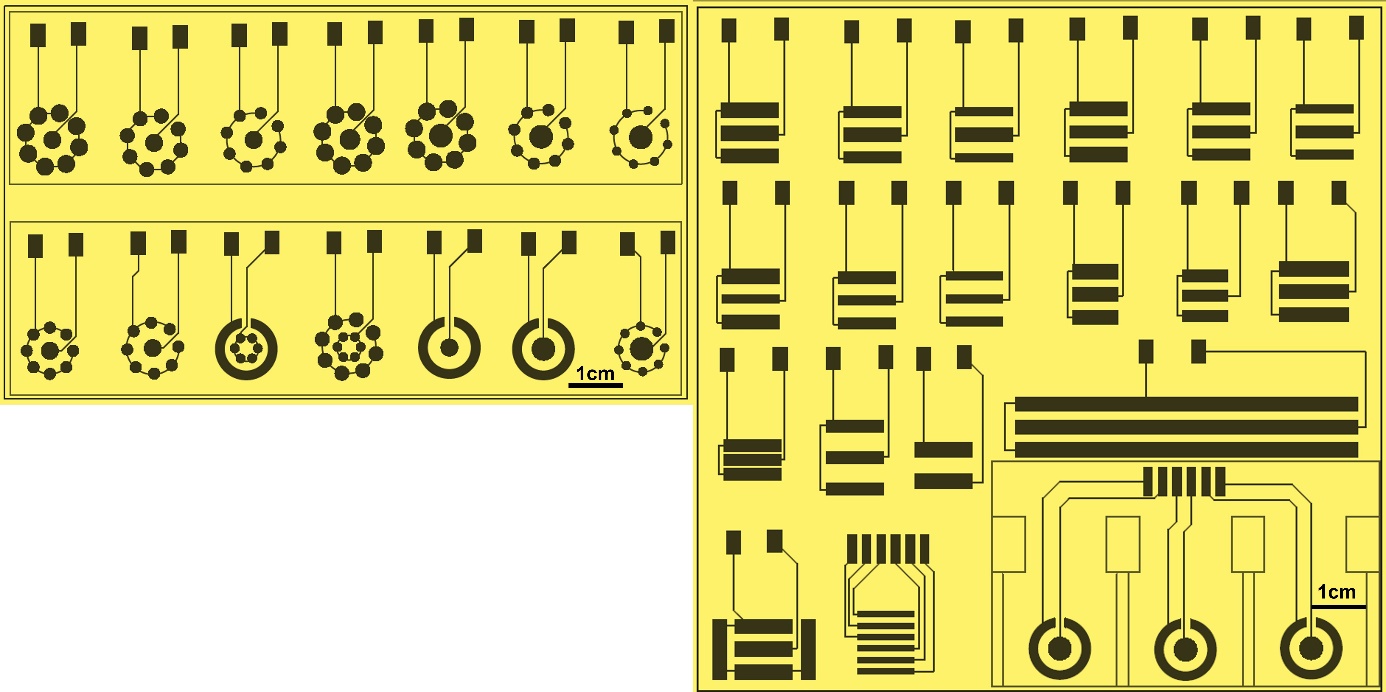


**Fig.S4**. Electrode arrays of different sizes and shapes. Sizes include the length, width, and distance of the anode (cathode). Shapes include circles and rectangles. The perception threshold of each design was measured. The threshold result indicates that the increase of the anode area, cathode area, and distance can reduce the voltage required to reach the threshold. The last electrode design causes the current to flow from one finger to the other, resulting in an uncomfortable tactile sensation that is not suitable for electrical stimulation applications.


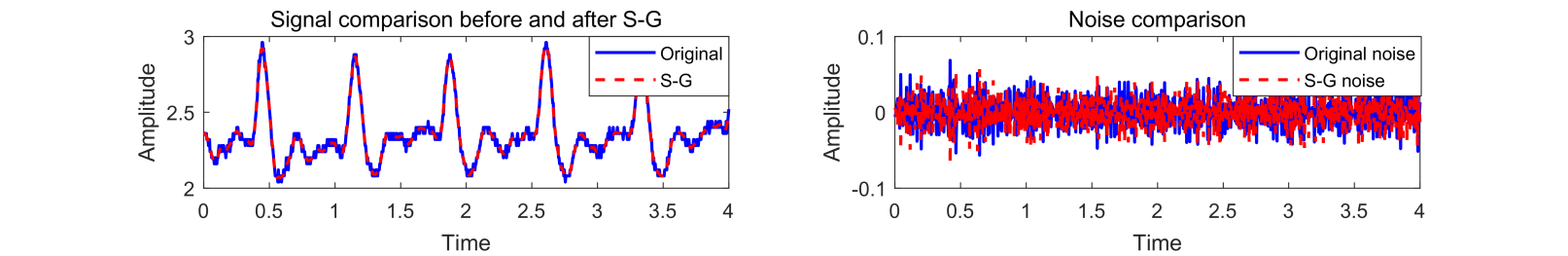


**Fig.S5**. Comparison of SNR before and after smoothing.

The SNR of the signal before and after smoothing the results are as follows:

The SNR of the signal before smoothing is: 19.94 dB

The SNR of the signal after smoothing is: 19.88 dB

The SNR of the signal after smoothing is the same or very close to the SNR of the signal before smoothing, indicating that the smoothing operation has little effect on the fidelity of the signal.

**Table S1.** Design of electrode types and parameters. The distance represents the central separation between the anode (red) and cathode (black).

| **Electrode type** | **Anode type** | **Cathode type** | **Anode area (mm^2^)** | **Cathode area (mm^2^)** | **Distance (mm)** |
| --- | --- | --- | --- | --- | --- |
| 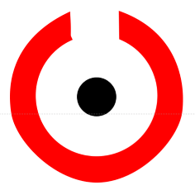 | Single | Single | 7 | 40 | 5 |
|  |  |  | 13 | 40 | 5 |
| 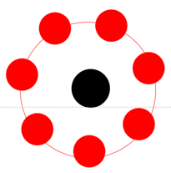 | Multiple | Single | 7 | 25, 39, 56 | 5 |
|  |  |  | 10 | 56 | 5 |
|  |  |  | 13 | 25, 56 | 5 |
|  |  |  | 7 | 25 | 4 |
|  |  |  | 13 | 14 | 4 |
| 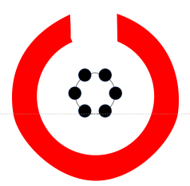 | Single | Multiple | 12 | 40 | 5 |
| 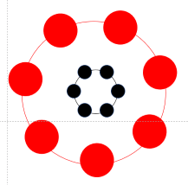 | Multiple | Multiple | 12 | 39 | 5 |
| 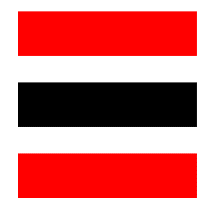 | Multiple | Single | 30 | 15, 20, 25 | 4 |
|  |  |  | 40 | 15, 20, 25 |  |
|  |  |  | 50 | 15, 20, 25 |  |
|  |  |  | 32 | 16 | 4 |
|  |  |  | 60 | 30 | 4 |
|  |  |  | 40 | 20 | 2.5, 5.5 |

**Table S2.** Parameter range of PRES.

| **Parameter** | **Range** |
| --- | --- |
| Waveform | Square wave(single-phase) |
| Voltage amplitude | 0-130V |
| Pulse width | 10-2000us |
| Frequency | 1-200Hz |

**Table S3.** Statistical table of intensity grading experiment.

| Name: Gender: Age: Dominant hand: | | | | | |
| --- | --- | --- | --- | --- | --- |
| I/mA | u/ms | N | Weak | Moderate | Strong |
| 1 | 0.1 | 1 |  |  |  |
| 1 | 0.1 | 5 |  |  |  |
| 1 | 0.1 | 10 |  |  |  |
| 1 | 0.4 | 1 |  |  |  |
| 1 | 0.4 | 5 |  |  |  |
| 1 | 0.4 | 10 |  |  |  |
| 1 | 0.8 | 1 |  |  |  |
| 1 | 0.8 | 5 |  |  |  |
| 1 | 0.8 | 10 |  |  |  |
| 3 | 0.1 | 1 |  |  |  |
| 3 | 0.1 | 5 |  |  |  |
| 3 | 0.1 | 10 |  |  |  |
| 3 | 0.4 | 1 |  |  |  |
| 3 | 0.4 | 5 |  |  |  |
| 3 | 0.4 | 10 |  |  |  |
| 3 | 0.8 | 1 |  |  |  |
| 3 | 0.8 | 5 |  |  |  |
| 3 | 0.8 | 10 |  |  |  |
| 6 | 0.1 | 1 |  |  |  |
| 6 | 0.1 | 5 |  |  |  |
| 6 | 0.1 | 10 |  |  |  |
| 6 | 0.4 | 1 |  |  |  |
| 6 | 0.4 | 5 |  |  |  |
| 6 | 0.4 | 10 |  |  |  |
| 6 | 0.8 | 1 |  |  |  |
| 6 | 0.8 | 5 |  |  |  |
| 6 | 0.8 | 10 |  |  |  |

**Table S4.** Statistical results of the subject experiment.

| Current (mA) | Pulse width (ms) | Pulse number | Weak | Moderate | Strong |
| --- | --- | --- | --- | --- | --- |
| 1 | 0.1 | 1 | 8 | 1 | 1 |
| 1 | 0.1 | 5 | 10 | 0 | 0 |
| 1 | 0.1 | 10 | 8 | 2 | 0 |
| 1 | 0.4 | 1 | 8 | 2 | 0 |
| 1 | 0.4 | 5 | 8 | 1 | 1 |
| 1 | 0.4 | 10 | 7 | 1 | 2 |
| 1 | 0.8 | 1 | 5 | 4 | 1 |
| 1 | 0.8 | 5 | 9 | 0 | 1 |
| 1 | 0.8 | 10 | 7 | 1 | 2 |
| 3 | 0.1 | 1 | 5 | 5 | 0 |
| 3 | 0.1 | 5 | 2 | 5 | 3 |
| 3 | 0.1 | 10 | 6 | 3 | 1 |
| 3 | 0.4 | 1 | 0 | 5 | 5 |
| 3 | 0.4 | 5 | 0 | 7 | 3 |
| 3 | 0.4 | 10 | 1 | 3 | 6 |
| 3 | 0.8 | 1 | 0 | 7 | 3 |
| 3 | 0.8 | 5 | 0 | 5 | 5 |
| 3 | 0.8 | 10 | 0 | 5 | 5 |
| 6 | 0.1 | 1 | 2 | 6 | 2 |
| 6 | 0.1 | 5 | 2 | 5 | 3 |
| 6 | 0.1 | 10 | 1 | 6 | 3 |
| 6 | 0.4 | 1 | 1 | 4 | 5 |
| 6 | 0.4 | 5 | 0 | 5 | 5 |
| 6 | 0.4 | 10 | 0 | 5 | 5 |
| 6 | 0.8 | 1 | 2 | 2 | 6 |
| 6 | 0.8 | 5 | 1 | 7 | 2 |
| 6 | 0.8 | 10 | 1 | 2 | 7 |

**Table S5.** Raw data of the force calibration experiment.

| I/mA | width/ms | N | F/ N | F/ N | F/ N | F/ N | Average/ N |
| --- | --- | --- | --- | --- | --- | --- | --- |
| 1 | 0.1 | 1 | 0.20 | 0.16 | 0.11 | 0.21 | 0.17 |
| 1 | 0.1 | 5 | 0.23 | 0.16 | 0.14 | 0.35 | 0.22 |
| 1 | 0.1 | 10 | 0.12 | 0.27 | 29 | 0.56 | 0.31 |
| 1 | 0.4 | 1 | 0.18 | 0.27 | 27 | 0.44 | 0.29 |
| 1 | 0.4 | 5 | 0.21 | 0.20 | 36 | 0.31 | 0.27 |
| 1 | 0.4 | 10 | 0.11 | 0.34 | 36 | 0.43 | 0.31 |
| 1 | 0.8 | 1 | 0.39 | 0.24 | 25 | 0.28 | 0.29 |
| 1 | 0.8 | 5 | 0.24 | 0.28 | 44 | 0.4 | 0.34 |
| 1 | 0.8 | 10 | 0.83 | 0.26 | 53 | 0.62 | 0.56 |
| 3 | 0.1 | 1 | 0.22 | 0.44 | 39 | 0.51 | 0.39 |
| 3 | 0.1 | 5 | 0.59 | 0.21 | 55 | 0.53 | 0.47 |
| 3 | 0.1 | 10 | 0.49 | 0.33 | 68 | 0.58 | 0.52 |
| 3 | 0.4 | 1 | 0.66 | 0.37 | 53 | 0.56 | 0.53 |
| 3 | 0.4 | 5 | 0.54 | 0.68 | 49 | 0.53 | 0.56 |
| 3 | 0.4 | 10 | 0.77 | 0.59 | 57 | 0.63 | 0.64 |
| 3 | 0.8 | 1 | 0.61 | 0.28 | 60 | 0.63 | 0.53 |
| 3 | 0.8 | 5 | 0.73 | 0.40 | 65 | 0.66 | 0.61 |
| 3 | 0.8 | 10 | 0.60 | 0.48 | 69 | 0.75 | 0.63 |
| 6 | 0.1 | 1 | 0.45 | 0.50 | 52 | 0.61 | 0.52 |
| 6 | 0.1 | 5 | 0.76 | 0.65 | 53 | 0.66 | 0.65 |
| 6 | 0.1 | 10 | 0.85 | 0.71 | 73 | 0.75 | 0.76 |
| 6 | 0.4 | 1 | 0.76 | 0.59 | 43 | 0.54 | 0.58 |
| 6 | 0.4 | 5 | 0.57 | 0.54 | 65 | 0.64 | 0.6 |
| 6 | 0.4 | 10 | 0.94 | 0.71 | 78 | 0.77 | 0.8 |
| 6 | 0.8 | 1 | 0.73 | 0.42 | 64 | 0.65 | 0.61 |
| 6 | 0.8 | 5 | 0.84 | 0.71 | 76 | 0.89 | 0.8 |
| 6 | 0.8 | 10 | 1.01 | 0.103 | 93 | 1.03 | 1 |

**Movie S1.**

**Demonstration of the threshold testing process**. Use the finger of the right hand to control the gradual increase in the stimulation intensity until the fingers of the left hand feel slight pressure. The oscilloscope displays each voltage increase.

**Movie S2.**

**Human-computer interaction interface of pulse condition reconstruction.** Use finger to touch the button on the screen to control the pulse reconstruction. The oscilloscope displays the voltage waveform of the PRES output to the fingers of the left hand.
